# 

**Published:** 1832

**Authors:** 


### PUBLISHER'S NOTICE.

The publisher of the Western Journal is happy to inform his patrons, that the course which the Journal has hitherto pursued, of knowing no friends, and recognizing no enemies, will still be rigidly adhered to. For the last six years, no pains have been spared to render it a neutral territory upon which individuals of every sect and party might meet with a certainty of having their rights respected and their just pretensions admitted. The public may rest assured that the Editors, under a presumption that in a world where so many sources of discord are at work, every individual has grievances enough of his own to attend to, will never be provoked, either by insult or injury, to prostitute to private malice or personal revenge, the pages of a work confessedly devoted to the interests of medical science.

E. DEMING.

